# Hyaluronan – A Functional and Structural Sweet Spot in the Tissue Microenvironment

**DOI:** 10.3389/fimmu.2015.00231

**Published:** 2015-05-15

**Authors:** James Monslow, Priya Govindaraju, Ellen Puré

**Affiliations:** ^1^Department of Biomedical Sciences, University of Pennsylvania, Philadelphia, PA, USA

**Keywords:** hyaluronan, remodeling, matrix, homeostasis, pathogenesis

## Abstract

Transition from homeostatic to reactive matrix remodeling is a fundamental adaptive tissue response to injury, inflammatory disease, fibrosis, and cancer. Alterations in architecture, physical properties, and matrix composition result in changes in biomechanical and biochemical cellular signaling. The dynamics of pericellular and extracellular matrices, including matrix protein, proteoglycan, and glycosaminoglycan modification are continually emerging as essential regulatory mechanisms underlying cellular and tissue function. Nevertheless, the impact of matrix organization on inflammation and immunity in particular and the consequent effects on tissue healing and disease outcome are arguably under-studied aspects of adaptive stress responses. Herein, we review how the predominant glycosaminoglycan hyaluronan (HA) contributes to the structure and function of the tissue microenvironment. Specifically, we examine the evidence of HA degradation and the generation of biologically active smaller HA fragments in pathological settings *in vivo*. We discuss how HA fragments versus nascent HA via alternate receptor-mediated signaling influence inflammatory cell recruitment and differentiation, resident cell activation, as well as tumor growth, survival, and metastasis. Finally, we discuss how HA fragmentation impacts restoration of normal tissue function and pathological outcomes in disease.

## Introduction

In the 80 years that have passed since hyaluronan (HA - also known as hyaluronic acid or hyaluronate) was first isolated and purified from the vitreous humor of the eye (1), the perception of this structurally seemingly simple molecule has changed dramatically. From simple beginnings, and being thought of merely as a “space-filler,” our understanding of its role grew slowly at first, steadily gathered steam and has now entered its exponential phase. HA is now recognized as a molecular powerhouse with critical roles in homeostasis, pathological disease onset, progression, and recovery or decline. This is none more so evident than in the number of review articles of which the biological role of HA has been the focus over the last few years alone [2012-2014 nearly 40 reviews, including an entire edition dedicated to its role in cancer (2)]. It is well established that native HA matrix found in homeostasis plays important biomechanical and biophysical roles as a hydrated cushioning agent and/or molecular filter in connective tissue, joints, and skin (3, 4). Furthermore, increased HA accumulation is a hallmark of almost all diseases in which inflammation and/or fibrosis occur, especially tumor growth and metastasis (2, 4–10). Importantly, HA polymer length (and thus its molecular weight, MW) plays a significant part in the nature of its interactions with the extracellular matrix (ECM), cell surface receptors (including its major receptor, CD44) on both resident and recruited cells, and influences how cells in tissue respond to extracellular cues under these conditions (4, 10–15). The existing literature clearly pinpoints that the MW characteristics of HA are important determinants of its biological activity, through *in vitro* and *in vivo* studies describing how exogenously added HA of different MW affects cellular signaling and function (4, 8, 11, 12, 16–19). There, however, has been limited work to elucidate the distribution of varying sizes of endogenous HA in the tissue in question, the alterations to HA MW that occur during disease progression and how these HA fragments change the biomechanical and biophysical properties of the tissue *in vivo*. Nor have many of the reports where exogenous HA was added, elucidated how this effected the size distribution of endogenous HA, and over time, its effects on tissue architecture and cellular signaling that translate to either recovery of homeostasis or progression of disease. This is especially important in the context of cancer progression, as the effects of altering HA MW may have varying and opposing effects depending on the origin of the cancer, the tissue in which it resides, and the stage of the disease (20). The recent findings in the naked mole rat that suggest a link between the animals’ resistance to cancer and the extraordinarily high MW (HWA) HA in its tissues have brought this subject into the limelight (21). For the above reasons, we have confined this review to focus on (i) a summary of the existing knowledge about HA MW distribution *in vivo* under homeostasis and disease, (ii) mechanisms responsible for alterations in HA MW and the occurrence of these mechanisms in pathological settings, and (iii) the opposing effects of HMW-HA versus HA fragments on ECM function, receptor-mediated cellular signaling and disease outcome.

## HA Molecular Weight Distribution in Homeostasis and Disease

### HA molecular weight – why do we care?

Hyaluronan is a polysaccharide of repeating units of d-glucuronic acid and *N*-acetyl-glucosamine. This highly charged, hydrophilic molecule is among the largest polysaccharides in nature, and in mammals one of the simplest with regards to structure. It is the major, non-proteinaceous component of the ECM, structurally distinct from other glycosaminoglycans (GAGs) in that it is unmodified (i.e., non-sulfated) and linear [non-branching (22)]. In its most common, homeostatic, and native form, HA polymer chain length exists as a HMW molecule, with sizes commonly above 1000 kDa. In this form, HMW-HA possesses biophysical properties that serve as a lubricant to hydrate tissue and create a matrix that sequesters growth factors and cytokines (23). It is uniquely synthesized at the plasma membrane with the completed polymer extruded to the extracellular space by the hyaluronan synthase enzymes (HASs). Increased HAS synthesis and HA accumulation are hallmarks of many pathological conditions (24). HMW-HA is degraded *in vivo* by hyaluronidases (Hyals), a family of enzymes that hydrolyze HA chains into intermediate (medium MW, MMW) or short (low MW, LMW) fragments (18). Changes in HA synthesis and degradation in part mediate the biochemical and rheological alterations to reactive matrices that occur during disease progression. Under certain pathological conditions, the extent of HA fragmentation is greatly enhanced, causing significant changes in the distribution and size of biologically active HA products, including the accumulation of HA oligomers [<10 kDa or <20 monomers – oligo-HA (25, 26)]. Collectively, these bioactive HA fragments serve to interact with cells and influence behavior in different ways to HMW-HA (27–31).

### HA MW distribution in health versus disease – it is the small (HA) things that matter

A correlation of increased HA levels in the pathological setting is now par for the course. However, understanding the MW distribution of HA *in vivo*, how it varies between different tissues, and how the ratio of HMW-, MMW-, LMW-, and oligo-HA changes during disease progression is also paramount when developing treatment regimens that target HA. Surprisingly, measurements of HA MW distribution *in vivo* have only occasionally been investigated; these are summarized in Table S1 in Supplementary Material.

Upon review of the literature, it became clear that there was no consensus for what was termed HMW- versus MMW-, LMW- and oligo-HA. To better understand and compare the roles of HA of different sizes under various biological settings going forward, we, for the purpose of this review, categorize the various MW forms of HA as follows; HMW-HA (>1000 kDa), MMW-HA (250-1000 kDa), LMW-HA (10-250 kDa), and oligo-HA (<10 kDa). These groups are by no means distinctly distributed; in many settings, HA MW is polydisperse, encompassing more than one size category. In contrast, specific properties of HA are in certain instances associated with a defined and narrow spectrum of its MW (12).

A total of 65 studies reported analysis of HA MW in an array of tissues including skin, brain, eye, prostate, blood, circulating leukocytes, synovial tissue and fluid, cartilage, amniotic fluid, lymphatics, kidney, aorta, gums, lung and lung fluid, heart, larynx, liver, cervix, skeletal muscle, and urine across a variety of species (see Table S1 in Supplementary Material for references). Nineteen of the studies analyzed HA MW under homeostatic conditions exclusively. Surprisingly, we only found eight studies that analyzed HA in the context of cancer. The remaining studies reported HA size in a number of pathological settings, including cardiovascular disease (atherosclerosis and vascular injury), arthritis (rheumatoid and osteoarthritis), liver disease (septic shock and chronic liver fibrosis), vanishing white matter disease, skeletal ischemia, lung disease (asphyxia, cigarette smoke exposure, asthma, fibrosis, ischemia, and hypertension), skin wounding/healing, kidney disease, development, pregnancy, inflammation, and aging. HA exists in a HMW form under homeostatic conditions in almost all of the tissues where it was analyzed, with subtle yet possibly significant differences depending on the tissue and species (1000-7000 kDa). Notably, increased HA fragmentation was evident under pathological conditions, occurring in both inflammatory and fibrotic diseases. HA MW analysis in lung and skin pathologies had been more extensively analyzed compared to other tissues. A small amount of HA was detected in lungs under homeostatic conditions, found predominantly in the HMW form. Following insult or injury, a dramatic increase in total HA as well as fragmentation yielding LMW-HA species was observed. Comparatively, under homeostatic conditions, skin contained a greater amount of HA, though still present in a HMW form. Following injury (by wounding or exposure to UVB radiation), HA was detected in either a LMW or MMW form (ranging from 100 to 350 kDa). As wounds healed, HA MW gradually transitioned back to its native HMW form after 28 weeks. Two reports analyzed the effects of aging in the context of wound healing and found decreased Hyal activity and delayed wound repair and restoration of HMW-HA in aged animals (mouse and rat). HA, HAS, and Hyal enzymes have been implicated in a variety of cancers. HA size has been reported in prostate, lung, brain, larynx, liver, colon, and urine (bladder) cancer. Increased HA fragmentation was observed in the prostate (1 of 2 reports), urine, larynx, brain tumor cyst fluid, and colon cancer compared to their normal counterparts, with no observed/reported change in MW distribution in lung or liver cancer. Interestingly, the presence of oligo-HA *in vivo* was only reported in five studies, two of which were under homeostatic conditions in the aorta and urine, one in the interstitial fluid of patients with colon cancer, and the remaining two studies in vascular tissue following injury. Collectively, such limited data, therefore, make it challenging to generalize and suggest how to manipulate HA with the goal of altering disease progression. Furthermore, changing HA MW in the milieu in one specific tissue may then not be translatable to the treatment of carcinogenesis and other pathological settings in different tissues.

## Physiologic and Pathophysiologic Mechanisms of HA MW Modification *In vivo*

### Is synthesis important?

Accurate measurements of HA MW profiles may be in short supply, but there is a substantial body of work focusing on the molecular processes that govern HA MW and the methods by which HA fragments accumulate. Under normal homeostatic conditions HA metabolism is carefully controlled to maintain physiological concentration in tissues. Furthermore, changes in HA synthesis and/or degradation are hallmarks of an ongoing pathological process (32). The three mammalian HAS enzymes (HAS1–3) synthesize and secrete HA polymers of different length; HA secreted into the culture media by stable transfectants revealed that HAS1 makes MMW-to-HMW-HA (200-2000 kDa), whereas HAS2 is responsible for only HMW polymers (>2000 kDa). HAS3 produces HA in the LMW-to-MMW range [100-1000 kDa (33)]. Stimuli such as cytokines [IL-1β, TNFα (34–36), and IL-15 (37)] and growth factors [TGF-β (36, 38, 39), PDGF (39, 40), HB-EGF (41), and EGF (42)] can regulate HAS expression at the transcriptional level. Furthermore, HAS activity has more recently been shown to be controlled by direct phosphorylation (43, 44), O-GlcNAcylation (45), and ubiquitination (46). The availability of UDP-sugar precursors (constituents of the HA disaccharide subunit) is also a rate-limiting step for HA production (47). HAS2 also has a natural antisense transcript at its gene locus (HAS-AS1) that can stabilize HAS2 mRNA (48).

Collectively, there are, therefore, many cellular mechanisms that can regulate HA levels in tissue at the stage of synthesis. However, there is currently no evidence suggesting that any of these factors, or others, alter the ability of the HAS enzymes to modify the length of HA polymer they produce. HA MW distribution, and specifically the accumulation of smaller polymers, appears to lie solely in mechanisms specifically controlling its degradation, with HAS enzymes only able to replenish existing extracellular HA reservoirs.

### HA turnover, catabolism, and the control of HA fragmentation

Extracellular HA can exist in a number of distinct pools that may have implications for HA turnover in homeostasis. Furthermore, any changes in the processes involved in the transition of HA from one pool to another could potentially affect its biological roles in the surrounding tissue. First, HA is synthesized as a HMW polymer that is retained as a pericellular coat, via retention by the HAS enzymes or receptor-mediated binding of HA following release of nascent chains from the synthases. Alternatively, HA can be released from the pericellular matrix and incorporated as an integral component of the ECM. Some may also be released as a soluble form into interstitial fluids or the circulation. In each case, HA is subject to subsequent degradation and either internalized and recycled by the resident cells or removed via the lymph. Under homeostatic conditions, HA has a high turnover rate, with as much as one third degraded to LMW fragments and replaced, each day. Lymphatic vessels drain considerable amounts of HA via receptor-facilitated uptake (utilizing receptors such as HARE, LYVE-1, and layilin), after which it is predominantly cleared in the liver. A small proportion is cleared by the kidneys (~10%) and only 1-2% is excreted in the urine (3, 49, 50). In tissues containing high amounts of native HA (skin, cartilage, and joints), a significant amount is degraded locally, by processes involving HYAL-mediated cleavage and receptor-aided internalization, via CD44 and RHAMM (49, 51, 52).

Hyaluronan fragmentation can occur via enzymatic or non-enzymatic processes. Enzymatic cleavage of HA by Hyal involves the hydrolysis of β-1-4 linkages in the HA chain (13). Six genes for Hyals have been identified in the human genome (Hyal-1–4, PH-20, and HYALP1), although only five of these encode protein products (not HYALP1) and of which only four can catabolize HA [not Hyal-3 (49)]. Hyal-1 cleaves HA over a wide MW range, down to oligo-HA fragments of only four or six saccharides in length (53). In contrast, Hyal-2 appears to cleave polydisperse HMW-HA to 20 kDa fragments (54), although the lower limit of HA size that Hyal-2 can digest is still unclear. Both intracellular (by Hyal-1) and extracellular (by Hyal-2) degradations of HMW-HA are CD44-dependent (55). PH-20 degrades polydisperse HA to oligo-HA (including HA disaccharides), but its expression is almost exclusively limited to sperm, where it degrades HA in the cumulus layer of oocytes to facilitate fertilization (56). Reactive oxygen/nitrogen species (ROS/RNS) are also capable of non-enzymatic HA depolymerization and fragmentation. This is a non-selective process, resulting in HA fragments of various lengths (57–60).

Under homeostatic conditions Hyal-1 is expressed in the major parenchymal organs, such as the liver, kidney, spleen, and heart, at low levels in lung, skeletal muscle, and placenta, and is also detectable in plasma and urine. In comparison, Hyal-2 is highly expressed in most tissues. Interestingly, neither isoform has been detected in the brain (13). Hyal-1 exists as a 56 kDa glycoprotein present in tissues and plasma and as a proteolytically processed 45 kDa form that is only found in plasma (12). Whether these two forms cleave HA to dissimilar fragments or have distinct preferences for HA polymers of different MWs remains to be elucidated. Hyal-1 is active at an acidic pH (53), suggesting that at a cellular level it may reside in lysosomal compartments. Hyal-2 shares many of the characteristics associated with Hyal-1; it has a MW of 55 kDa, also exists as a proteolytically processed smaller form and is optimally active at an acidic pH. In contrast, Hyal-2 contains a glycosylphosphatidylinositol (GPI) linkage, thus tethering it to plasma membrane surfaces. There is a consensus that Hyal-2 may also localize to lysosomes, although there is some conflicting evidence that suggests this may not be the case (12). It is also unclear if membrane-tethered Hyal-2 is involved in releasing cell-associated HA from the pericellular environment in order that it can be integrated into the ECM. Hyal-3 is somewhat of an anomaly; strong transcriptional expression has been detected in bone marrow, testes, and kidney, although no changes in HA accumulation were observed in Hyal-3-deficient mice (61). To date, no activity has been detected *in vivo*, although Hyal-3 may contribute to HA metabolism and fragmentation by altering the activity of Hyal-1 (62).

### Evidence for Hyal expression, activity, and Hyal- or ROS-dependent HA fragmentation during disease progression *in vivo*

Any augmentation to Hyal expression, activity, or receptor-mediated lymphatic drainage has the potential to result in the accumulation of smaller bioactive HA fragments in tissue, and this has proven to be a hallmark of a variety of pathological conditions *in vivo*. Indeed, the genetic disorder mucopolysaccharidosis IX arises due to a mutation of Hyal-1. This mutation attenuates the ability of the enzyme to degrade HA, resulting in increased levels of HA in plasma and elevated storage of mucopolysaccharide in lysosomes (63). Table S1 in Supplementary Material documents changes in HA MW distribution in a variety of biological settings, which in some instances have been correlated with, or arise as a result of alterations in Hyal content. This is certainly true in inflammatory disease; platelet-derived Hyal-2 increases the accumulation of HA fragments that in turn stimulate monocytic IL-6 and IL-8 production and downstream inflammatory responses in the local milieu (64). Furthermore, human CD14^+^ monocytes from normal as well as myelomonocytic lineages from leukemia patients express Hyal on their cell surface, thereby possessing the potential to degrade HA in the circulation as well as upon their recruitment to sites of disease (65). Increased Hyal activity, together with increased levels of LMW-HA has been reported in highly inflammatory atheromatous plaques during cardiovascular disease (66). Diabetes also correlated with increased Hyal expression in vascular tissue, with increased HA fragmentation (67). Conversely, increased deposition of MMW–HMW-HA led to severe cardiac dysfunction in Hyal-2-deficient mice (68). A variety of lung disorders have been examined for correlations between Hyal levels and HA fragmentation. Hyal-1 expression is increased in a model of pulmonary hypertension leading to accumulation of HA fragments (69). Airway smooth muscle cells from asthmatic or chronic obstructive pulmonary disease (COPD) patients have a reduction in average HA MW (250 kDa) versus healthy controls (>700 kDa) that correlates with increased expression of Hyal-1 (70). Furthermore, increased Hyal-2 expression, together with decreased HAS2 expression has been reported in patients with COPD (71). Generation of ROS, combined with increased Hyal-2 activity also increases HA fragmentation (72). ROS-dependent HA fragmentation was also supported in two other separate studies, where exposure to cigarette smoke and subsequent ROS generation reduced pulmonary native HA (>500 kDa) to LMW-HA [70 kDa (73)], whereas pulmonary ischemia was associated with increased accumulation of LMW- and MMW-HA fragments [30-495 kDa (74)]. Interestingly, this latter study showed that HA fragmentation resulted solely from ROS activity, and not via Hyal degradation. In a model of skin injury, UVB irradiation of organotypic epidermal cultures induced Hyal, HAS, and CD44 expression, leading to an accumulation of LMW-HA fragments (75). HA fragmentation has also been recognized as a biological marker for rheumatoid arthritis (76). Indeed, TNFα-stimulated synovial fibroblasts from arthritic mice show increased levels of LMW-HA (77).

Alterations in Hyal expression, activity, and HA fragmentation have also been reported in some oncogenic settings. Increased expression and activity of Hyal-1 and Hyal-2 were observed in a study of patients with colorectal cancer, with the highest activity found in the advanced stages of the disease (78). Overexpression of Hyal-1 also promoted mammary tumor growth and increased tumor angiogenesis (79). Increased Hyal activity together with the accumulation of HA fragments promoted pancreatic tumor cell motility (30), and the tumor cell line H460M (derived from human lung cancer) was also reported to produce high levels of Hyal, although not its own HA (80). Hyal expression has been found at elevated levels in other malignancies, including head and neck (81), prostate (82), brain (83), and urinary tract (84). Further reports suggest that increased Hyal levels might serve as a diagnostic marker for the onset and progression of bladder and epithelial ovarian cancer (58, 85, 86). However, evidence exists that contradict these findings. A study of patients with endometrial cancer indicated that tumor tissues had elevated HA levels and correlated with lower expression of Hyal-1 and Hyal-2 compared to healthy controls (87). Decreased Hyal expression has also been reported in squamous cell head and neck carcinoma (88) as well as lung cancer (89). Furthermore, increased Hyal activity by genetic manipulation or intravenous administration suppressed tumor growth in models of colon and breast carcinoma, respectively (90, 91). In a separate study, ablation of HA in the tumor stroma by intravenous injection of Hyal decreased intratumoral fluid pressure and consequentially increased drug penetration in a model of pancreatic ductal carcinoma (92).

Collectively, current evidence suggests that HA synthesis and degradation are delicately balanced. The likelihood exists that alterations in either HA synthesis or degradation can have profound consequences on the other, which may account for opposing outcomes in disease progression. Indeed there is some evidence that supports this notion. Overexpression of HAS1 in prostate cancer was shown to be anti-tumorigenic; however, overexpression of both HAS1 and Hyal-1 increased HA fragmentation that in turn promoted tumor cell proliferation and metastasis (93). Furthermore, Hyal-1 expression in cancer cells themselves functioned as both a tumor promoter and tumor suppressor in prostate carcinoma (82). In a separate study in breast cancer, expression of antisense HAS2 (ASHAS2) increased accumulation of HMW-HA, while simultaneously causing the downregulation of Hyal-2. Combined, this inhibited the initiation and progression of primary and metastatic tumor progression (94).

## The Opposing Effects of Native HA Versus HA Fragments on ECM Function, Receptor-Mediated Signaling, and Disease Outcome

### HMW-HA - keeping tissues in check?

As discussed, HA in its native state is found in a HMW form (>1000 kDa) that influences normal homeostatic functions in a variety of ways. HMW-HA has the ability to trap large amounts of water, thus possessing biophysical properties that serve to lubricate, hydrate, or space-fill tissues such as joints and connective tissue (13, 95). Its hydrophilic attributes also allow it to act as a molecular sieve and affect fluid absorption rates to and from tissue through changes in its concentration (96). HMW-HA possesses biomechanical properties, and none more so is this evident than during development. The maturing embryo is surrounded by a soft, hydrated matrix, rich in HMW-HA. Soft matrices are commonly considered to inhibit cellular adhesion and proliferation. Uniquely, the HA-rich microenvironment during development facilitates growth and development of tissues, including neuronal growth (97), limbs (98), blood vessels, and the heart (99). The presence of HMW-HA is critical to normal embryogenesis, as targeted deletion of HAS2 results in embryonic lethality, due to abnormal heart development (99). These distinct biomechanical properties of HA in soft tissue have recently been confirmed *in vitro*, where HA-rich soft substrates, as opposed to collagen-rich substrates of the same degree of stiffness but lacking HA, promoted cell spreading, focal adhesion and stress-fiber formation, and normal cell function (100). This might explain how embryonic cells are able to adhere, proliferate, and differentiate under soft conditions during development. It is interesting to speculate whether one may extend this hypothesis to wound healing; one of the first events in the formation of granulation tissue (for example in skin wounds) is an accumulation of HA in a soft fibrin matrix. The HA may serve to facilitate cell migration in the early stages of wound healing, prior to the onset of fibroproliferation, and the generation of a stiffer substratum as a result of increased matrix deposition. A HA-rich fibrin clot may enable swift tissue remodeling and healing in the face of initially soft conditions.

High molecular weight-HA-specific signaling has been shown to result in favorable outcome on cell and tissue function in adult tissues in response to environmental cues. In *in vitro* wound healing models, the incorporation of HMW-HA into collagen gels enhanced gel contraction, vascular smooth muscle cell (VSMC) cell spreading, filopodia formation, and pericellular accumulation of collagen fibers via the HA receptor CD44 (101). HMW-HA also promoted actin stress-fiber arrangement, lamellipodia formation, and cell migration (but not proliferation) in VSMCs (102). Inhibition of CD44 blocked HA–CD44–RhoA-mediated events, with the exception of migration, whereas inhibition of RHAMM (another HA receptor) and downstream Rac signaling only inhibited HA-mediated migration (102). HMW-HA also enhanced myocardial repair when transplanted simultaneously with bone marrow mononuclear cells in the heart following myocardial infarction. The HMW-HA provided a favorable microenvironment for transplanted cell adhesion and proliferation, leading to reduced inflammation and cardiomyocyte apoptosis, as well as increased angiogenesis and cardiac performance (103). HMW-HA also increased CD44- and NF-κB-dependent SNAIL2 expression leading to increased fibroblast invasion (104). Indeed, the role of HMW-HA in response to injury has been studied quite extensively. In the skin, HMW-HA improved permeability barrier function in aged epidermis via CD44-dependent mechanisms (105). This may in part be attributed to HMW-HA–CD44-dependent mechanisms that control keratinocyte differentiation (106). HMW-HA also enhanced excisional wound contraction compared with saline-treated controls (107). In a diabetic wound-healing model, this was associated with enhanced angiogenesis, TGF-β, and transglutaminase II expression, restoration of cyclin B1/Cdc2 complex and increased mechanical strength (108). Independently, HMW-HA was shown to mitigate astrocyte activation *in vitro* and *in vivo* leading to a reduction in scarring (109). Furthermore, daily subcutaneous administration of a HMW-HA formulation (HYAL-BV 5200) inhibited neointimal formation and macrophage recruitment following balloon catheter-induced vascular injury in cholesterol-fed rabbits (110). Our group reported that the tissue response to vascular injury was CD44-dependent. HMW-HA inhibited mesenchymal cell cyclin D1 expression and subsequent cell proliferation via a CD44-dependent and Skp2-dependent mechanism (111). HMW-HA may also control the response to injury by reducing VSMC apoptosis mediated via TLR4, CD44, and downstream PI3K signaling (112).

Many of the protective/healing effects associated with HMW-HA in response to injury can be attributed to its suppressive effects on the inflammatory response. HMW-HA (1600 kDa) completely blocked monocyte and neutrophil infiltration and MIP-2 and TNFα induction in a model of sepsis-induced lung injury, thus attenuating the injury response (113). T-cell-mediated liver injury, as well as the release of pro-inflammatory cytokines TNFα, IFNγ, MIP2, and IL-4, was also inhibited by administration of HMW-HA (114). HMW-HA increased SDF1b-induced CXCR4 signaling and cell motility, increased vessel sprouting, and angiogenesis. This process was again HA–CD44 dependent, with CD44 physically interacting with CXCR4 in the presence of the CXCL12 ligand (115).

High molecular weight-HA is also able to enhance immunosuppression via binding to the surface receptor TLR4 leading to an increase in the release of the immunosuppressive cytokine IL-10 (116). Interestingly, this immunosuppressive effect has recently been suggested as a mechanism in infectious disease; HMW-HA impaired virus phagocytosis by macrophages and thus increased viral survival within the blood (117).

### HMW-HA and cancer progression

The protective effect of HMW-HA is also evident in tumorigenesis. Treatment with HMW-HA inhibited post-chemotherapy tumor growth in a human colon carcinoma xenograft model in NSG mice (118). HMW-HA antagonized the pro-inflammatory effects of IL-1β-treated chondrosarcoma cells, decreasing COX2, MMP-1, and MMP-13; promoting Akt; and suppressing MAPKs and NF-κB signaling, via PPARγ-dependent signaling (119). HMW-HA also inhibited migration of fibrosarcoma cells. Interestingly, this effect was fibrosarcoma cell line-specific and did not occur in any of the other cancer cell lines that were tested (120). Conversely, overexpression of HAS2 (presumably leading to increased accumulation of HMW-HA) facilitated the reversion of cancer cells to a stem cell-like state via Twist and TGF-β signaling and thus promoted tumor cell survival (121). It is, however, unclear if this survival was due directly to HMW-HA signaling, or due to further degradation of the accumulated HMW-HA and pro-oncogenic signaling as a result of an accumulation of HA fragments.

### Oligo-HA - the gloves are off

Whereas evidence points to HMW-HA as protective and facilitating in the restoration of homeostasis in pathological settings, the effects of oligo-HA could not be more further removed. Many of the effects exerted by oligo-HA in pathological conditions occur via receptor-mediated signaling in immune cells leading to the promotion or protraction of inflammation. Oligo-HA induces the phenotypic maturation of human monocyte-derived dendritic cells (DCs) and the production of inflammatory cytokines IL-1β, TNFα, and IL-12. Interestingly this was not mediated via the HA receptors CD44 or RHAMM and was partly mediated indirectly via TNFR (122). Subsequently, DC maturation by oligo-HA was found to be mediated by its binding to TLR4 (and downstream p38/p42/44 MAP-kinase pathways). This was confirmed *in vivo*, as oligo-HA induced DC emigration from skin, as well as their phenotypic and functional maturation in the spleen (123). Oligo-HA administration to resting monocytes increases the expression of the scavenger receptor (CD36), uptake of oxidized LDL and their transendothelial migration. This particular response was CD44-dependent and mediated in part via the PKC pathway (124). Together, these data suggest that CD44 is directly implicated in prolonged inflammatory responses in many auto-inflammatory conditions such as atherosclerosis. Furthermore, it suggests that CD44 may promote the conversion of macrophages to foam cells within lesions, leading to increased lesional lipid accumulation and immune cell content, conditions that favor lesion rupture, a risk factor for heart attack and stroke.

Oligo-HA receptor-mediated cell responses are not limited to the immune system. Endothelial cells (ECs) in particular are impacted by oligo-HA. Oligomers of 6, 8, and 10 disaccharides (but not 4 subunits) promoted EC proliferation and VEGF secretion (125). Increased EC proliferation in response to oligo-HA also increased tube formation, upregulation of the adhesion proteins ICAM and VCAM as well as the release of pro-inflammatory cytokines (126). *In vivo*, oligo-HA has been reported as one of the predominant mechanisms by which ECs respond to injury, with these responses mediated via oligo-HA–TLR4-dependent mechanisms (127). However, ECs also express ICAM and CD44, both of which can bind oligo-HA and potentially mediate cellular function (128, 129). Indeed, oligo-HA was shown to induce rapid upregulation of immediate-early genes c-fos, c-jun, jun-B Krox-20, and Krox-24, responsible for angiogenesis, in a CD44-dependent manner. Additionally, immediate-early gene signaling was not sufficient to induce EC proliferation and was only induced upon long-term treatment with oligo-HA (129). Oligo-HA is also capable of inducing pro-inflammatory signals in chondrocytes. IL-1β treatment induced inflammatory signaling pathways that were mediated via oligo-HA–CD44 activation, leading to an increase in NF-κB, TNFα, IL-6, MMP-13, and iNOS, as well as the CD44 receptor itself (130).

A certain degree of inflammation and, therefore, the generation of some oligo-HA is a normal part of the body’s response to insult. That being said, excess oligo-HA has often been shown to be detrimental to healing, causing protracted inflammation thus favoring disease progression. In contrast, the effects of oligo-HA on resident mesenchymal cells suggest that it may facilitate tissue recovery and healing. Sustained delivery of oligo-HA by nano-particles increased elastin synthesis and lysl oxidase expression in rat SMCs to facilitate aortic remodeling following injury (131). Topical application of oligo-HA promoted keratinocyte proliferation and increased skin thickness and barrier function, in a CD44-dependent manner (105). Wound healing models have also revealed that administration of oligo-HA accelerates wound healing by promoting wound closure, the accumulation of M1 and M2 macrophages, release of TGF-β (132), enhanced angiogenesis, lymphogenesis, and ECM deposition (133). Oligo-HA-accelerated wound closure was both CD44- and RHAMM-dependent. Interestingly, although fibroblast proliferation was increased, myofibroblast differentiation within the granulation tissue did not change (132). Protective roles for oligo-HA have also been reported in cardiovascular disease. Together with TGF-β, oligo-HA, but not larger HA polymers (20, 200, or 2000 kDa), cooperatively enhanced elastin matrix regeneration in VSMCs (134), whereas oligo-HA administration protected against neointima formation in the aorta following balloon catheter injury *in vivo* (135). Oligo-HA can also upregulate hsp72 expression by enhancing the activation of HSF1 in response to hyperthermia in synovial cells, which acts as a protective mechanism by suppressing cell death (136). A separate recent study suggests a separate biochemical mechanism by which oligo-HA may facilitate healing. The covalent transfer of heavy chains (HCs) from inter-α-inhibitor (IαI) to HMW-HA via the protein product of tumor necrosis factor-stimulated gene-6 (TSG-6) forms a HC–HMW-HA complex, a pathological form of HA that promotes the adhesion and retention of leukocytes to HA matrices (137). The transfer of HCs to HMW-HA is a reversible event mediated by TSG-6, whereas HC transfer to oligo-HA is irreversible. Treatment of HA–HC-rich synovial fluid from arthritic mice with oligo-HA and TSG-6 irreversibly shuttled HCs from pathological, HMW HC–HA to the oligo-HA. This suggests that oligo-HA could thereby facilitate the restoration of HA matrices in the inflamed joint to its normal, unmodified state, by removing HCs from HMW-HA, through more efficient clearance of HCs from tissue (138).

### Oligo-HA and cancer progression

The current literature is divided upon whether oligo-HA (as well as LMW–MMW-HA as discussed later) promotes or suppresses tumor growth and metastasis. Complexities arise due to the wide and more varied effects that oligo-HA appears to have on tumors, compared to the effects it has on non-transformed cell types in other pathological conditions. This no doubt stems from the simple fact that tumors can develop in almost all tissues, from different cellular origins, and as a result of different mutations/environmental cues, to the point where you are in effect, looking at the role of one molecule (oligo-HA) on an ever increasing number of functionally and pathologically distinct, tumors. A number of these opposing effects are discussed below.

Oligo-HA induces CXCR7 expression in a TLR4-dependent manner, leading to the proliferation of W3 papillary thyroid carcinoma cells *in vivo* (28). In another independent study, oligo-HA preferentially stimulated a physical association between CD44 with TLR2, TLR4 and the recruitment of MyD88 and actin filament-associated protein 110 (AFAP-110), leading to NF-κB translocation and downstream expression of IL-1β and IL-8 in MDA-MB231 breast cancer cells. Combined, this promoted tumor cell invasion (139). Oligo-HA also upregulated the expression and acute phosphorylation of c-met, leading to proliferation, differentiation, and invasion of human chondrosarcoma cells. This effect was dependent on oligo-HA–CD44 interaction and signaling, and not observed with any other HA MW size (29). Similarly, oligo-HA, but not LMW- or HMW-HA, induced MMP-9, -13, and uPAR in Lewis lung carcinoma (LLC) tumor cells, thus facilitating matrix remodeling and tumor cell migration. Interestingly, this response was not dependent on the interaction of HA with CD44, RHAMM, or TLR4 (140). More complex roles of oligo-HA with CD44 have also been reported in pancreatic carcinoma. Oligo-HA, generated by Hyal degradation, enhanced the cleavage of CD44 and its release into the ECM, which in turn enhanced tumor cell motility. This phenomenon was abrogated upon inhibition of HA–CD44 binding. This suggests that tumor cells enhance their own CD44 cleavage via Hyal activity and oligo-HA generation to promote their own motility, tumor invasion, and metastasis (30). In contrast, oligo-HA has the ability to kill many types of tumor cells by triggering apoptosis, while leaving normal cells unaffected. In an interesting extension of these findings, chemo-resistant tumor cells became drug-sensitive when treated in combination with oligo-HA (141). Indeed, this may be one mechanism by which oligo-HA inhibits the growth of B16F10 melanoma growth *in vivo* (142). This phenomenon has been observed in other tumor models and appears to be a response that is dependent on oligo-HA and CD44 interaction. Oligo-HA, competitively blocked binding of endogenous HMW-HA to CD44, consequently attenuating downstream signaling to the PI3K/Akt cell survival pathway, leading to inhibited tumor growth and apoptosis (143, 144). Furthermore, oligo-HA suppressed glioma growth *in vivo*, in part through inhibiting recruitment of progenitor BRCP+ stem cells (145). There is also evidence that oligo-HA abrogated cell-associated matrices and HA retention via CD44 in osteosarcoma, resulting in apoptosis and importantly, suppression of the formation of lung metastases (31).

### MMW-HA and LMW-HA - caught in the middle?

Low molecular weight-HA and MMW-HA fragments are frequently detected as polydisperse fractions with overlapping MW distributions. They could be considered intermediate fractions, and arise due to partial fragmentation due to varying concentrations of Hyal, ROS/RNS, as well as the availability of cleavage sites depending on HA interactions with its receptors and other ECM proteins. There is also the possibility that these fractions contain nascent HA that has not yet reached its full length. Taking this into account, MMW-HA and LMW-HA have roles that overlap with either HMW-HA or oligo-HA.

Low molecular weight–MMW-HA has been reported to facilitate the differentiation of many mesenchymal cells that are activated as a normal response following injury, including chondrocytes (146), fibroblasts [together with their expression of growth factors FGF-2 and KGF (147)], keratinocytes (148), and VSMCs. VSMC differentiation was associated with increased collagen deposition (149). LMW-HA improved dermal excisional would repair, associated with increased expression of CD44 and RHAMM and deposition of type-III collagen in aged mice (150). A separate study also showed improved age-related skin function, when HA was administered to patients with skin atrophy in a CD44-dependent manner (151). Topical administration of LMW-HA also acts as a scavenging agent following xenobiotic treatment (and ROS generation), promoting wound healing in excisional and incisional wound models (152). In the lung, LMW-HA protected against porcine pancreatic elastase-induced bronchoconstriction (153). Its protective effect against elastase was confirmed in a second model where aerosolized LMW-HA blocked experimental emphysema induced by intra-tracheal administration of elastase (154). Conversely, administration of LMW-HA exacerbated ozone-induced airway hyper-reactivity in a CD44-dependent manner, also in the lung, whereas treatment with HMW-HA protected against ozone injury (155). LMW-HA, via TLR4-mediated receptor binding, induced neutrophil apoptosis via an IFNβ, TRAIL/TRAILR-dependent mechanism, thus protecting against prolonged inflammation following injury (156). LMW-HA has also been reported to induce apoptosis of myeloid cells via CD44-dependent, tyrosine kinase signaling (157). Its protective effects have additionally been reported in the liver and intestine, by preventing hepatocellular apoptosis via NF-κB (158) and the expression of murine β-defensin 3 (an ortholog of human β-defensin 2) via TLR4, respectively (159).

Low molecular weight-HA and MMW-HA promote inflammation through direct and indirect signaling mechanisms. Directly, polydisperse LMW–MMW-HA increases inflammatory gene expression and decreases anti-inflammatory signaling in macrophages by downregulating surface expression of A2aR, via CD44 and PKC (160). LMW–MMW-HA binding to HARE has also been shown to activate pro-inflammatory NF-κB signaling (161). Conversely, LMW–MMW-HA activates the innate immune response via TLR2 and MyD88 (162). LMW-HA can mobilize leukocytes but not hematopoietic progenitor cells to the circulation (163) and increase NO production in primary macrophages (164). Importantly, the activation of elicited, versus resident peritoneal macrophages by LMW-HA have distinct requirements. Both cell types produce pro-inflammatory cytokines (including IL-12) in response to LMW-HA via LMW-HA–CD44 signaling; however, resident macrophages require adhesion-dependent priming to respond to LMW-HA (165). Other pro-inflammatory cytokines released following LMW-HA stimulation include MIP1-α, MCP-1, RANTES, and Crg-2 (166). LMW–MMW-HA is also a potent stimulator of eicosanoids (including induction of COX2 and PGE2 production via ERK1/2 p38 and JNK signaling) in primary human monocytes and murine wild-type bone marrow-derived monocytes. This activation was also dependent on a HA–TLR4/MyD88 pathway (167). LMW-HA can also influence macrophage polarity. M0 (undifferentiated) and M2 (pro-fibrotic) macrophages can be switched to an M1 (pro-inflammatory) phenotype after a short period of stimulation with LMW-HA (167). However, prolonged exposure to LMW-HA can induce the M2 phenotype (168).

Indirectly, LMW–MMW-HA can stimulate the production of pro-inflammatory stimuli from other cell types. Lung epithelial cells, chondrocytes, liver ECs, and VSMCs release a number of pro-inflammatory cytokines in response to LMW-HA via its binding to CD44 or TLR4, including TNFα, IL-1β, MMP-13, and iNOS (169–174). In VSMCs, LMW-HA also stimulated cell proliferation and migration via CD44 through ERK1/2 and RhoA signaling (173, 175). One study also found that TLR4 interacts with CD44 in response to LMW-HA and together act as a brake in LMW-HA-induced lung inflammation (176). *In vivo*, LMW-HA promoted splenocyte proliferation, macrophage activation, while suppressing angiogenesis in chicken embryos (177). LMW-HA is critical in the induced fetal growth response to uterine ischemia/reperfusion via TLR4 (178). LMW-HA also decreases the rate of early wound contraction in skin (107), but increased total number of recruited macrophages in the granulation tissue (132).

### LMW–MMW-HA and cancer progression

In the tumor microenvironment (TME), LMW–MMW-HA has the potential to influence cancer cells, stromal cells, ECs, and infiltrating inflammatory cells. Specifically, LMW-HA facilitates tumor cell adhesion and migration. In fibrosarcoma, this was shown to be dependent on LMW-HA–RHAMM binding, which influenced downstream FAK and ERK1/2 signaling (179). LMW-HA enhanced proliferation (though MAPK and c-fos signaling) and adhesion of LM8 murine osteosarcoma cells with increased MMP-2 secretion. Adhesion in these cells was shown to be dependent on CD44 (180). In fact, LMW-HA–CD44-dependent signaling has been reported in a number of carcinoma cells to activate NF-κB signaling via a Ras-PKCζ-IκB cascade (181), and thus may be one of the many ways in which LMW-HA promotes tumor progression by the activation of cell proliferation. However, much like what has been found for oligo-HA in the TME, LMW–MMW-HA can also inhibit growth of some tumors. MMW-HA stimulated iNOS and subsequent NO production and apoptosis in DCs *in vivo* in glioma. This process was dependent on HA–CD44 interaction and suggests that HA in gliomas may contribute to immunosuppression by promoting apoptosis of infiltrating immune cells (182). In contrasting findings to those above, LMW-HA inhibited colorectal carcinoma growth *in vivo*, by inhibiting tumor cell proliferation via Akt signaling (183). The TME was also affected; LMW-HA induced immunity against the carcinoma by stimulating DC migration, proliferation, and the release of IL-12 and IFNγ, while simultaneously decreasing their release of immunosuppressive IL-10. Interestingly, these responses occurred in a CD44 and TLR4-independent manner (184).

### CD44 - an important regulator of HA-mediated signaling in cancer?

Among the receptors involved in HA signaling, CD44 is the most abundant, expressed in almost all tissues and across nearly every cell type. Additionally, this type I transmembrane protein is able to bind almost all HA MW species, with the exception of oligo-HA fragments smaller than six saccharides in length (16). Combined, these two contributing factors can arguably account for the variety of different functions of HA–CD44 interactions *in vivo*, any of which could have important outcomes on tumorigenesis.

As well as being bound to the plasma membrane, CD44 can exist as a cleaved, matrix-associated fragment and as a soluble protein and can independently affect cellular function (185, 186). CD44 also exists as a number of splice variants, which are commonly expressed in tumor cells (6). These variants encode additional segments in the membrane proximal region of the extracellular domain that can be differentially glycosylated (187, 188). The CD44 cytoplasmic domain is known to be required for HA binding, the formation and retention of pericellular matrix, and CD44-mediated endocytosis of HA (189, 190). Furthermore, cell adhesion via HA binding can be regulated in part by variable glycosylation of its CD44 extracellular domain, as increased glycosylation inhibits HA recognition (191). These post-translational modifications (N and O glycosylation) of CD44 also affect its ability to signal and shed from the cell surface; however, CD44 cleavage can also occur via a glycosylation-independent mechanism via MMP cleavage (192). Interestingly, murine M0 macrophages when stimulated to induce pro-inflammatory M1 polarization (with LPS/IFNγ), upregulate their CD44 expression and ability to bind HA. Conversely, M0 macrophages polarized to the anti-inflammatory M2 phenotype (with IL-4) also upregulate CD44 expression but with no increase in HA binding. This difference was a consequence of the loss of chondroitin sulfation on CD44 in M1s and conversely an upregulation of chondroitin sulfation on CD44 in M2s (193). It is as-yet unclear if this dynamic physiological regulation of hyaluronan binding also influences the phenotypic differences between the two cell types and the inflammatory state of the TME. Nevertheless, the ability to alter macrophage polarization via CD44 offers a potentially new mechanism to target the inflammatory response *in vivo*, in the context of tumor progression as well as other inflammatory conditions, such as wound healing, lung injury, and atherosclerosis.

CD44 knockout mice are viable and fertile with a modest phenotype; progenitor cell egress from the bone marrow is slightly impaired (194). However, under pathological conditions, deletion of CD44 has profound effects on tissue architecture, signaling, and disease outcome. In a model of non-infectious lung injury, CD44-null mice succumb to unremitting inflammation with impaired clearance of neutrophils, persistent accumulation of LMW-HA, and impaired activation of TGF-β. This phenotype was partially restored by reconstitution with CD44^+^ bone marrow-derived cells (195). In contrast, we previously reported that CD44 promotes auto-inflammatory disease progression in a mouse model of atherosclerosis. CD44 expression correlated with increased lesional macrophage and HA content and VSMC activation (196). Furthermore, CD44 on both bone marrow-derived and non-bone marrow-derived cells was important; CD44 on leukocytes in part promoted the disease via enhancing macrophage and T-cell recruitment to lesions *in vivo*. Leukocyte to EC adhesion and transmigration was also CD44-dependent as was macrophage activation. CD44 on VSMCs also promoted their migration and proliferation (197). Interestingly, we also reported that CD44 is selectively upregulated in athero-prone regions, and CD44 signaling impacts gene expression profiles in the vasculature, including those genes involved with focal adhesion formation, ECM deposition, inflammation, and angiogenesis (198).

CD44 is known to facilitate the rolling and adhesion of circulating leukocytes on the endothelium, and subsequent transendothelial migration (14, 199–201). Targeted inhibition of HA–CD44 binding using a synthetic peptide inhibits leukocyte adhesion and trafficking *in vivo* (202). HMW-HA–CD44 binding in calveolin-enriched microdomains (CEMs) in ECs also promotes barrier function, via the recruitment of c-met, Tiam1, Rac1, dynamin 2, and cortactin to CEMs and their redistribution to areas of cell–cell contact (203, 204). Any alterations to HA–CD44 interaction could, therefore, potentially alter the recruitment of immune cells or the intravasation of tumor cells to the blood stream or extravasation to tissue during metastasis. Administration of LMW-HA suppresses A2aR (a negative regulator of inflammation) via CD44 binding and downstream PKC signaling following lung injury *in vivo* (160). The HA receptor TLR4 has been shown to interact directly with CD44 in order to limit LMW-HA-induced lung inflammation *in vivo* (176). Antisense CD44 inhibited HA binding, tumor growth, and metastasis of colorectal carcinoma cells to the liver (205). Furthermore, peptide inhibition of CD44–HA binding significantly reduced seeding and tumor growth of intravenously introduced B16-F10 melanoma cells in lungs in a model of metastasis (206). Indeed, HA–CD44 interaction has been implicated in the growth of a number of cancers (6). The growth-inhibitory and tumor-suppressive effects of p53 act in part via its ability to bind to a non-canonical sequence in the CD44 promoter, thus inhibiting CD44 expression and downstream tumor-promoting signaling in breast cancer cells (207). Furthermore, a separate study found that human miRNAs miR-373, and miR-520c suppressed CD44 expression, leading to the promotion of breast tumor cell migration and invasion *in vitro* and *in vivo* (208). The standard form of CD44 (CD44s) and CD44v6 are involved in breast cancer cell adhesion and motility via interactions with HA (209). This increased cell motility perhaps occurs as a result of modulation of CD44 into clusters by HMW-HA on the plasma membrane (210). CD44 clusters facilitate cell binding and internalization of HA thus enabling invasion from tumor masses into the surrounding ECM (211). Observation of neuroblastoma cells revealed that these CD44 clusters localized to filopodia and focal bleb-like protrusions in neuroblastoma cells that enabled migration and invasive growth into brain tissue (212). In another study, chondroitin sulfate E fragments enhanced CD44 cleavage and tumor cell motility upon degradation. Much like LMW-HA, these degradation products modulated tumor cell adhesion and migration by binding to CD44 (213). In a separate finding, the non-coding 3′UTR of CD44 was found to act as a binding site for miRNAs that targeted the genes for Col1α1 and fibronectin. Overexpression of the CD44 3′UTR in MDA-MB231 cells antagonized the effects of the miRNAs on their specific targets and upregulated collagen and fibronectin expression that in turn enhanced tumor cell migration and metastasis *in vivo* (214).

Stromal expression of ECM proteins and GAGs is increased in activated fibroblasts in the TME and is thought to promote tumor cell migration. Thus, targeting CD44 signaling in stromal cells in the TME may also provide a separate avenue to target tumorigenesis. Indeed, CD44 has been implicated in stromal cell function in a number of pathological settings. CD44 facilitates the healing response, by promoting fibroblast infiltration, proliferation, myofibroblast differentiation, and ECM deposition and remodeling following myocardial infarction (215, 216). Fibroblast migration is mediated by CD44-dependent TGF-β activation that promotes stress-fiber formation and directional migration (217). The HA receptor RHAMM may also facilitate cell migration via the regulation of CD44–Erk1/2 complexes at the cell surface (218). Cell proliferation via CD44 is dependent upon the MW of the HA ligand. In VSMCs, HMW-HA binding to CD44 selectively inhibits the GTP loading of Rac and Rac-dependent signaling to cyclin D1 (thereby inhibiting proliferation), whereas LMW-HA binding to CD44 selectively stimulates ERK activation and ERK-dependent cyclin D1 expression [thus promoting proliferation (175)]. Interestingly, HA binding to CD44 increases as a function of HA size. Half maximal saturation is reached with a HA MW of only 30 kDa. Reversible binding was confined to oligo-HA fragments (<10 kDa), with interactions essentially irreversible with large polymers [>30 kDa (219)]. The accumulation of oligo-HA in tumors, combined with increased CD44 expression, may, therefore, be a mechanism to activate alternate, pro-tumorigenic signaling pathways as a means to bypass and eventually overcome the non-reversible protective signals stemming from HMW-HA–CD44 binding.

## Conclusions, Caveats, and Perspectives

In revisiting the literature, we found that little was known about how HA MW distribution changes *in vivo* during disease, especially when compared with the number of studies that reported changes in HA content in pathological settings. Much of what is known about HA MW in disease *in vivo* has been extrapolated from *in vitro* cell culture experiments and only a handful of early articles where it was analyzed *in vivo*. Furthermore, the limited number of studies, which we found that reported the occurrence of oligo-HA in tissue (five in total) question the pathophysiologic relevance of oligo-HA, and its effects on cell function *in vivo*. Many studies have tested the biological activity of HA (from oligo-HA to HMW-HA) as an exogenously added ligand to cells in culture (with some extending these experiments *in vivo*), and its effects on cellular signaling, gene/protein expression, and cell behavior; some revisited how it changed endogenous HA, including its localization, matrix organization, and cross-linking and turnover, but very few specifically analyzed its effects on endogenous MW distribution. One could argue that HA MW analysis is not a straightforward technology. There are no commercial kits that allow HA MW profiles to be analyzed in a high throughput fashion. Additionally, care has to be taken to first extract HA from its link proteins, HCs, and other ECM molecules with which it forms higher ordered structures, while simultaneously taking measures to prevent loss of the smaller oligo-HA fragments during purification. The commercially available kits for measuring HA concentration (ELISA and ELISA-like assays) also vary, with some unable to detect HA polymers accurately at the extremes of the MW spectrum (220). On the other hand, thanks to a number of dedicated groups, a variety of reliable methodologies for HA MW analysis do exist, using combinations of size-exclusion chromatography (221), flat-bed polyacrylamide electrophoresis (222, 223), agarose gel electrophoresis (223–227), gas phase electrophoretic mobility molecular analysis [GEMMA (228)], or asymmetrical flow field fractionation with multi-angle light scattering (229, 230), for continuous HA size profiling. Consequently, we anticipate the increased use of these robust methodologies to monitor HA size in various pathological settings *in vivo* in future studies.

**Figure 1:**
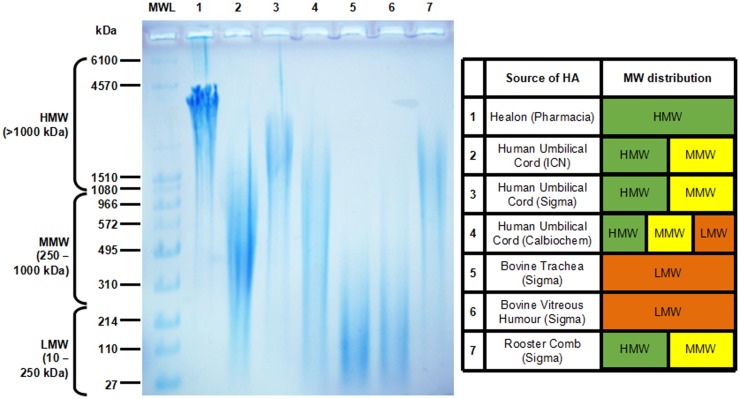
**Agarose gel electrophoresis showing the molecular weight (MW) distribution of commercially available HA (left) and charted for comparison (right)**. Molecular weight HA ladder (MWL) was purchased from Hyalose (combined mega, high, and low ladders). HA MW is divided into high (HMW >1000 kDa), medium (MMW, 250-1000 kDa), and low (10-250 kDa).

High molecular weight HA and oligo-HA exist as distinct pools of HA with unique biological properties at the opposite ends of the HA MW spectrum. On the other hand, LMW-HA and MMW-HA are frequently detected as polydisperse fractions that often overlap. When searching for articles related to LMW-HA and MMW-HA in the literature, we found that the MW range was not always reported, instead being replaced with the average MW for the sample. This was found to be very common in earlier studies, and where LMW-HA or MMW-HA fractions were added exogenously as extracellular cues to examine their effects on cell function. These LMW and MMW fractions were purchased from various companies, with the HA sourced from various tissues from different species. Those most commonly used included umbilical cord (human), rooster comb, trachea and vitreous humor (bovine), and a synthetic polymer of HMW-HA produced by Pharmacia (known as Healon). The polydisperse MW ranges of HA for these preparations are given in Figure 1. Umbilical cord HA was found to be commonly used in the literature as LMW-HA. We compared umbilical cord HA fractions from three separate vendors. Remarkably, the MW distribution of umbilical cord HA was hugely polydisperse and varied depending on the vendor, ranging from 30-1000 kDa for HA from ICN (MP Biomedicals) to 900-3500 kDa from Sigma, and 200-3000 kDa when obtained from Calbiochem. Two of the sources of umbilical cord HA (Sigma and Calbiochem) contained a significant proportion of HMW (>1000 kDa) HA. This high degree of variation in HA MW may account for some of the opposing data we found regarding the function of LMW-HA in the literature where umbilical cord HA had been used. We were only able to obtain single vendor samples for tracheal, vitreous humor, and rooster comb HA. The MW distribution of HA in these fractions in comparison were much narrower, with tracheal and vitreous humor HA in the 30-300 kDa range (LMW) and rooster comb at 600-3000 kDa (MMW-HMW). The synthetic, Healon HA could be considered very high, at 3500-4500 kDa). In order to better understand and compare the roles of HMW-HA versus HA fragments in future studies, non-overlapping HA MW samples with reduced polydispersity for each sized group will help define the often contrasting roles of HA on cell function. To this end, it appears that using umbilical cord HA may not be best suited for this purpose. In any case, HA from this source does not appear to be any longer available, nor is HA from bovine trachea. This leaves Healon, rooster comb HA, and vitreous humor HA as three sources, with non-overlapping MW profiles that can be confidently used to investigate the unique individual roles of different HA MWs on tissue function and disease outcome in future studies. This would be along with the use of LMW-HA, oligo-HA, and narrow-range HA preparations manufactured using unique bacterial fermentation technologies (spearheaded by Hyalose and now offered by other vendors) that are now readily available (231–233).

**Figure 2:**
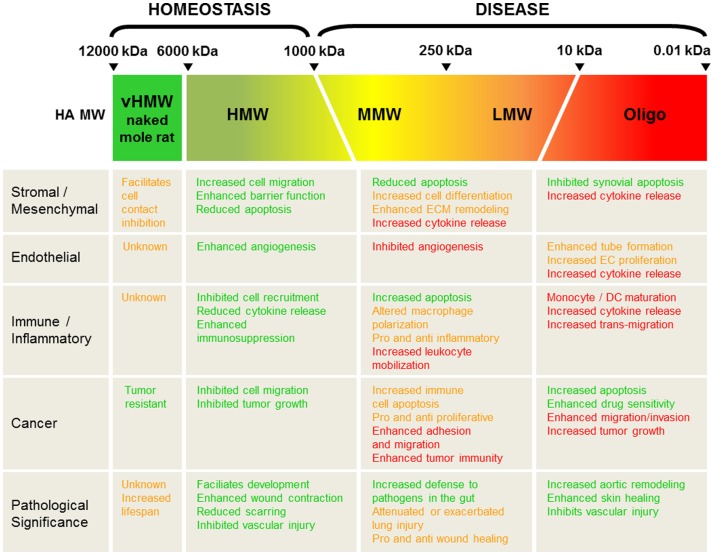
**Summary and pathological significance of HA size *in vivo***. HA molecular weight (MW) is divided into very high (vHMW 6000-12000 kDa; naked mole rat HA), high (HMW > 1000 kDa), medium (MMW 250-1000 kDa), low (10-250 kDa), and Oligo-HA (< 10 kDa). Green text highlights positive roles for each HA MW in tissue function and recovery, whereas red text favors pathological decline. Opposing and/or unclear tissue responses are designated in orange-colored text.

Depending on the pathological setting, HA fragmentation may be good or bad, pro- or anti-inflammatory, aid in tissue recovery or promote disease progression, as summarized in Figure 2. The complexity in part arises due to the number of different signaling mechanisms that result from HA itself acting as a ligand; it can mediate alternate and often opposing effects via different, yet often the same receptors. Furthermore, HA in tissue is often found as a polydisperse molecule, often covering a MW range from 20 to 2000 kDa with no one, specific length of HA polymer dominating during disease. It is, therefore, interesting that in its polydisperse state, HA can produce such contrasting signaling cues compared to what is exerted by native, HMW-HA in homeostasis. It is possible that changes in HA-mediated cellular signaling occur when a small percent of this polydisperse-fragmented pool passes a threshold that is enough to tip the balance and change the outcome of cellular function. Indeed, we have previously proposed that CD44 may act as a rheostat for cell proliferation through its ability to activate alternative signaling pathways via the binding of HMW-HA versus LMW-HA (22). As small pools of HA fragments have the propensity to do this, it is, therefore, conceivable that therapies to induce even possibly quite modest changes in the ratio of the active HA fragments would be enough to shift the balance of signaling in the favor of tissue repair and recovery. For example, small adjustments in increasing the ratio of HMW-HA to HA fragments may be enough to keep disease in check. On the other hand, there may be occasions where decreasing this ratio, and increasing the levels of specific HA fragments may also be beneficial. Importantly, the total concentration of HA fragments versus native, HMW-HA also needs to be measured and addressed; simply flooding the system with any size HA might be expected to cause detrimental effects to the tissue. Understanding, therefore, how HA MW distribution changes *in vivo* in different pathological settings, together with the shifts and trends that alter HA signaling, will be crucially important in deciding when and where to intervene to alter the course of disease progression.

It is clear that there is a substantial body of work that has investigated the biological and signaling roles of HMW-HA, MMW-HA, LMW-HA, and oligo-HA on cell function and disease outcome. Collectively, the data suggest that using HMW-HA may be a suitable course of action to aid tissue recovery and a return to homeostasis (Figure 2). This has certainly been the case for the treatment of arthritis (234). Other important avenues of research have pinpointed a pivotal role for HA in the regenerative properties of fetal tissues, which unlike their adult counterparts, heal without scarring (235), although the mechanisms by which this occurs remains to be elucidated. A less well-studied area is how MMW-HA, LMW-HA, and oligo-HA impact the biophysical and rheological properties of the tissue microenvironment or the biomechanical properties of HA in the pericellular and ECM. Going forward, these also offer important and exciting areas of investigation that may offer new therapeutic perspectives on targeting HA biology in tumor and disease progression and restoration of homeostasis.

## Conflict of Interest Statement

The authors declare that the research was conducted in the absence of any commercial or financial relationships that could be construed as a potential conflict of interest.

## Supplementary Material

The Supplementary Material for this article can be found online at http://journal.frontiersin.org/article/10.3389/fimmu.2015.00231

Table S1 **HA size in tissue *in vivo***. HA molecular weight (MW) is divided into High (HMW >1000 kDa), Medium (MMW 250–1000 kDa), Low (10–250 kDa), and Oligo-HA (<10 kDa).
